# Giant uterine artery pseudoaneurysm after a missed miscarriage termination in a cesarean scar pregnancy

**DOI:** 10.1186/1472-6874-14-89

**Published:** 2014-07-29

**Authors:** Yun Mou, Yuezhen Xu, Ying Hu, Tianan Jiang

**Affiliations:** 1 Department of Ultrasound, the First Affiliated Hospital, College of Medicine, Zhejiang University, Hangzhou, P. R. China; 2 Department of Ultrasound, Zhejiang Xinan Hospital, Jiaxing, P.R. China

**Keywords:** Uterus, Pseudoaneurysm, Ultrasound, Missed miscarriage, Cesarean scar pregnancy

## Abstract

**Background:**

Uterine artery pseudoaneurysms are dangerous and can lead to severe hemorrhage. We report an uncommon cause of a giant pseudoaneurysm in a missed miscarriage in a woman with a cesarean scar pregnancy.

**Case presentation:**

The patient was a 25-year-old Chinese woman with a missed miscarriage in a cesarean scar pregnancy. Curettage was performed under ultrasound monitoring. A uterine artery pseudoaneurysm measuring 71 × 44 × 39 mm was detected the next day by Doppler ultrasonography. While waiting for admittance to an advanced institution to undergo embolization treatment, the pseudoaneurysm ruptured spontaneously. The subsequent severe hemorrhage necessitated hysterectomy.

**Conclusion:**

A delay in diagnosis of uterine artery pseudoaneurysms may result from a long period between the curettage and follow-up examination. Ultrasound and Doppler ultrasonography should be performed repeatedly at short intervals to rule out them, especially in cesarean scar pregnancies. For a giant uterine artery pseudoaneurysm, interventional embolization might be the first treatment choice. If time allows, intra-operative ligation of the feeding vessels should be attempted before any decision to perform a hysterectomy is made. However, hysterectomy remains a possibility when severe bleeding occurs.

## Background

Uterine artery pseudoaneurysms are rare complications that can arise after repeated curettage, abortions, cesarean sections, uncomplicated vaginal deliveries or reproductive tract infections. They are dangerous and can lead to severe hemorrhage. The interval from pelvic surgery to the onset of symptoms is typically 1 week to 3 months [1-4]. This delay is supposed to be caused by a gradual increase in the size of the pseudoaneurysm caused by a characteristic pressure increment. The blood flow into the pseudoaneurysm is greater during systole than diastole. This leads to a gradual pressure build up and eventual rupture. It can be treated with hysterectomy with or without hypogastric artery ligation. In recent years, uterine artery embolization has become an accepted treatment method for this condition. The option depends on the patient’s reproductive desires and hemodynamic situation.

A literature search found three case reports of cesarean scar pregnancies complicated with a uterine artery pseudoaneurysm [5-7]. Here, it occurred in a patient with a missed miscarriage during a cesarean scar pregnancy and the lesion was the largest reported to date.

## Case presentation

The 25-year-old Chinese female patient had been amenorrheic for 2 months and was referred to the hospital because of painless vaginal bleeding that had lasted for 15 days. Four years ago she had a missed miscarriage at 9 weeks of gestation, which was managed by surgical curettage. Two years prior to presentation she had delivered a baby by elective cesarean. Twenty days before presenting at the hospital she had been diagnosed as 40 days pregnant based on elevated urinary levels of beta human chorionic gonadotropin (β-hCG).

**Figure 1:**
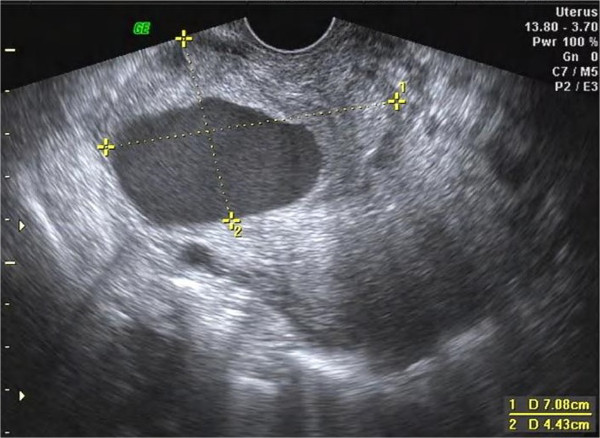
**Two-dimentional ultrasound image of the pseudoaneurysm.** The pseudoaneurysm is visible in the anterior wall of the lower part of the uterus. The wall varied in thickness and the luminal surface of the wall is uneven.

**Figure 2:**
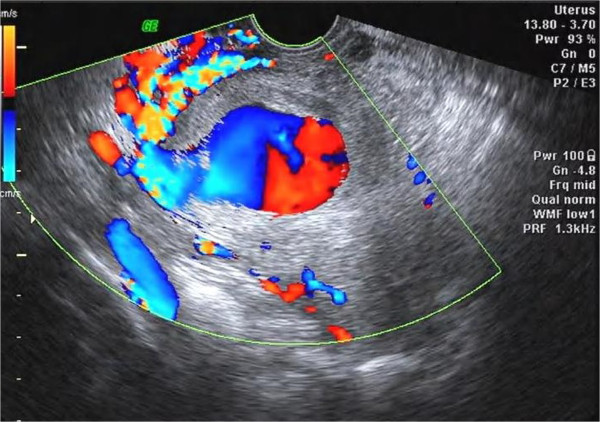
**Doppler ultrasonography of the pseudoaneurysm.** A swirl of colors, which represents the opening of the pseudoaneurysm and its supplying artery, is visible in this color Doppler ultrasonography image.

**Figure 3:**
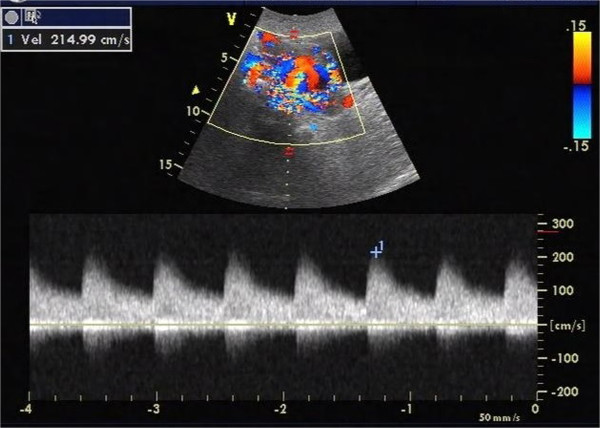
**Blood flow velocity within the supplying artery.** Pulsed Doppler ultrasonography showed that the velocity of blood in the supplying artery near the orifice of the pseudoaneurysm was as high as 215 cm/s.

The study was approved by the Institutional Review Board at the First Affiliated Hospital, College of medicine, Zhejiang University. The procedures were conducted according to the principles of the Helsinki Declaration. On presentation the vaginal bleeding was scanty and her serum β-hCG level was 1200 mIU/mL. Transvaginal ultrasonography showed that the uterus measured 106 × 64 × 60 mm. There was an echo-free area above the inner cervical os, measuring 43 × 23 mm, without any blood flow signal, yolk sac or embryo present. A mixed echo mass measuring 29 × 15 mm was detected in the uterine cavity but no blood flow signal could be found in it. The patient was diagnosed as having had a missed miscarriage in the cesarean scar region of the uterus and curettage was performed with ultrasound monitoring. During the procedure, massive bleeding (~600 mL) occurred but this was stopped with an intravenous injection of oxytocin and uterine massage. Chorionic tissue was aspirated and proven as such by histopathology. When the curettage was finished, the uterine cavity was revealed as a clear thin line by ultrasound and was considered normal. Vaginal packing was performed subsequently.At 18 hours after curettage, the serum β-hCG level was 1164 mIU/mL. A cystic lesion with an uneven wall in the lower part of the uterus measuring 71 × 44 × 39 mm was detected with gray-scale ultrasonography (Figure 1). Color Doppler ultrasonography showed a swirl of colors in the cystic lesion (Figure 2), which was connected to an artery via a narrow neck in its posterior wall. The peak velocity in the artery was as high as 215 cm/s (Figure 3).

**Figure 4:**
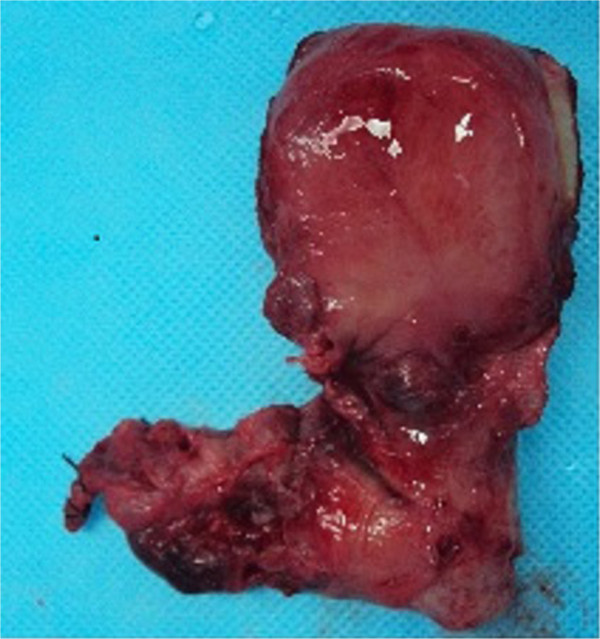
**Gross specimen of the uterine artery pseudoaneurysm.** The giant pseudoaneurysm (left) was ruptured, and its size was consistent with the ultrasonography findings.

The patient was examined by interventional radiologists; however, they lacked experience in treating such a giant lesion and thought that repeated treatment might be required. A persistently high serum β-hCG concentration suggested that there were retained chorionic villi present within the pseudoaneurysm. Thus, recanalization of the pseudoaneurysm by rapid recruitment of collateral vessels might occur even after arterial embolization. Repeated uterine curettage immediately after embolization, or methotrexate therapy, might decrease the likelihood of recanalization. Therefore, the patient sought admittance to an advanced institution for embolization treatment. However, while waiting to be admitted, massive bleeding occurred suddenly at day 10 and emergency surgery was performed.During the operation, a 60 × 70 × 50 mm pseudoaneurysm was located in the cesarean scar position (Figure 4). Ideally, the lesion would have been completely resected and the uterus repaired after ligation of the uterine artery feeding the pseudoaneurysm. However, the hemorrhage was immediately life threatening. Therefore, to stop the bleeding quickly and save the patient’s life a hysterectomy was performed. The wall of the pseudoaneurysm was composed of clotted blood, decidual tissue and chorionic tissue.

## Discussion

Uterine artery pseudoaneurysms are very dangerous and should be diagnosed as soon as possible. Potential causes include vascular injury following abortion, curettage, and pelvic surgery. Traumatic injury to the vessel wall causes wall incompetence and hemorrhage leading to a pseudoaneurysm. In this case, both two-dimensional and Doppler ultrasound scans were useful in detecting the pseudoaneurysm.

In two-dimensional ultrasonographic images, a pseudoaneurysm manifests as a hypoechoic mass and is thereby not easily differentiated from a hematoma or a true aneurysm. Color Doppler ultrasonography was helpful in this case as it demonstrated turbulent arterial flow with a to-and-fro pattern, connected to a parent artery by a narrow neck in the pseudoaneurysm. Blood flow into the mass during systole and away from the mass during diastole can be explained by the pressure gradient between a distended high-pressure pseudoaneurysm and the low pressure in the artery during diastole [8]. A true aneurysm manifests as a color-coded fusiform dilation of the parent artery and spectral analysis can demonstrate a typical arterial flow pattern. A simple hematoma does not reveal any color signal caused by turbulent blood flow. The wall of a pseudoaneurysm is formed by a peripheral thrombus. In this case, decidual tissue and chorionic tissue were also found in the wall. We diagnosed the pseudoaneurysm by ultrasonography at the day after curettage. Therefore, we think it is necessary for patients to undergo a Doppler ultrasound examination as a required postoperative investigation especially in cases of cesarean scar pregnancy. Repeating these examinations several times over a short time period might allow early diagnosis of a pseudoaneurysm, when it is still small in size.

In this case, the pseudoaneurysm was located in the cesarean scar. The wall of the pseudoaneurysm was very thin and at high risk of rupture, it should have been treated as rapidly as possible. In such cases, endovascular treatment is often the first-line therapy. Embolization can be achieved with coils, stents and injectable liquids [9,10], and offers the potential of preserving fertility for the patient. However, in the literature, the pseudoaneurysms treated successfully by this method were only 0.6–3.5 cm in diameter [11].

If time allows, and if embolization is not an option, intra-operative ligation of the pseudoaneurysm feeding vessels should be attempted prior to resorting to hysterectomy, especially in patients with low parity [12]. Ligation of the ascending branch of the uterine artery may successfully stop postabortal hemorrhage in approximately 90% of patients.

Another possible treatment method was direct injection of thrombin into the pseudoaneurysm [13], but no further experience has been reported in the literature. Thus, we lack knowledge on the scope of possible complications associated with this procedure, such as subsequent arterial thrombosis or allergic responses.

## Conclusions

A delay in diagnosis of uterine artery pseudoaneurysms may be caused by a long period between the curettage and follow-up examination. Ultrasound and Doppler ultrasonography are recommended to be performed repeatedly at short intervals to rule out them, especially in cesarean scar pregnancies. For a giant uterine artery pseudoaneurysm, interventional embolization might be the first treatment option when the diagnosis is made. Alternatively, if time allows, intra-operative ligation of the feeding vessels should be performed prior to any decision to resort to hysterectomy. However, hysterectomy remains a possibility when severe bleeding occurs.

### Consent

Written informed consent was obtained from the patient for publication of this Case Report and the accompanying images. A copy of the written consent is available for review by the Editor of this journal.

## Competing interest

The authors declare that they have no competing interests.

## Authors’ contributions

YM carried out the ultrasonography, participated in treatment of the patient and wrote the manuscript. YX assisted with the ultrasonography and followed up the treatment of the patient. YH participated in the design of the study and helped to draft the manuscript. TJ conceived of the study and participated in the diagnosis and in drafting the report. All authors have read and approved the final manuscript.

## Pre-publication history

The pre-publication history for this paper can be accessed here:

http://www.biomedcentral.com/1472-6874/14/89/prepub
